# Seroprevalence and associated risk factors of leptospirosis in slaughter pigs; a neglected public health risk, western Kenya

**DOI:** 10.1186/s12917-019-2159-3

**Published:** 2019-11-08

**Authors:** Jeremiah N. Ngugi, Eric M. Fèvre, Georgies F. Mgode, Mark Obonyo, Ginethon G. Mhamphi, Christina A. Otieno, Elizabeth Anne Jessie Cook

**Affiliations:** 1 Field Epidemiology and Laboratory Training Program, Kenyatta National Hospital, P.O. Box 22313, Nairobi, 00100 Kenya; 2 County Government of Taita Taveta, P.O. Box 1066-80304, Wundanyi, Kenya; 3 International Livestock Research Institute, Old Naivasha Road, PO Box 30709, Nairobi, 00100 Kenya; 4 Institute of Infection and Global Health, University of Liverpool, Leahurst Campus, Chester High Road, Neston, CH64 7TE UK; 5 Pest Management Centre, Sokoine University of Agriculture, Morogoro, Tanzania; 6 Ministry of Agriculture, Livestock and Fisheries, Directorate of Veterinary Services, Nairobi, Kenya; 7 School of Public Health, Moi University, Eldoret, Kenya

**Keywords:** Leptospirosis, Occupational exposure, Slaughterhouse workers, Microscopic agglutination test

## Abstract

**Background:**

Leptospirosis is a neglected zoonosis of public health importance transmitted through contact with contaminated soil, water or urine of infected animals. In pigs the disease is characterized by abortion, still births and weak piglets. A cross-sectional study was conducted in May to July 2018 to estimate the sero-prevalence of leptospirosis and factors associated with seropositivity in slaughter pigs. A questionnaire was used to collect information on animal demographics. Serum was tested for anti-leptospiral antibodies using microscopic agglutination test (MAT) with a panel of 8 serovars. Sera were considered positive for sero-reactivity at a MAT titre ≥1:40 against at least one serovar. Chi-square tests were used to measure the strength of association between the MAT test result and exploratory variables.

**Results:**

A total of 252 pig serum samples from seven slaughterhouses were tested for *Leptospira* antibodies by MAT. Of the 252 pigs sampled, 88.8% (244/252) were indigenous breeds; 55.6% (140/252) were female and 88.7% (220/252) were reared in extensive production systems. Eighty-three (32.9%; 83/252) sera samples tested positive on MAT against at least one serovar. Of the 8 serovars, the highest prevalence was recorded for serovar Lora 21.4% followed by Kenya 5.2%, Sokoine 3.6% and Grippotyphosa at 3.2%. Risk factors for leptospirosis seropositivity in pigs were: originating from farms with other types of livestock (OR 2.3; 95% CI 1.0–4.5) and mature pigs (OR 1.9; 95% CI 1.1–3.3).

**Conclusion:**

This study demonstrates that there is a high prevalence of leptospirosis positive pigs at slaughter in a small-holder livestock keeping region of the Lake Victoria basin. The potential for cross species transmission of pathogenic serovars is highlighted as well as the potential for occupational exposure to slaughterhouse personnel. Improvements in husbandry practices (confinement and rodent control) and public health education among slaughterhouse workers and other high-risk groups is recommended.

## Background

Leptospirosis is a bacterial zoonotic disease caused by pathogenic serovars of the genus *Leptospira* which were historically divided into two species; pathogenic *Leptospira interrogans* and saprophytic *Leptospira biflexa.* However, genetic classification has grouped *Leptospira* spp. into eight pathogenic genomospecies (*L. alexanderi, L. alstonii, L. borgpetersenii, L. interrogans, L. kirschneri, L. weilii, L. noguchii and L. santarosai*) [1–4]. In addition, *Leptospira* have been further classified serologically into more than 250 pathogenic serovars [5, 6]. Leptospirosis is transmitted directly through contact with urine or body fluids of infected animals or indirectly through water or soil contaminated with urine from infected animals. Domestic animals including pigs harbor leptospires in the kidneys and genital tracts where they can persist for a long period of time with intermittent shedding in urine. This acts as a source of infection to humans and other animals [7–9]. The level of susceptibility varies within the domestic species and each serovar tends to be maintained in a particular animal species [10]. Animals can be infected with serovars maintained by the same animal species or other animal species in the same geographical location [2].

Porcine leptospirosis has been reported most often in South East Asia and South America due to the favorable weather conditions for environmental survival and transmission of leptospires [11]. In a serological survey in Colombia using the microscopic agglutination test (MAT) in different animal species, a seroprevalence of 55.9% in pigs was reported [7]. Another study in fattening pigs in 5 provinces in Vietnam reported an overall seroprevalence of 8.17% by MAT [12]. In regions where pig management practices include vaccination against leptospirosis, the overall seroprevalence has been on the decline [1, 2]. This decline has also been attributed to improved housing since it limits animal-environmental interaction [1, 2]. A study in pig farms in Greece reported a seroprevalence of 17.8% by MAT [13]. In Sicily Italy, a study of free-roaming semi-wild black swine demonstrated leptospires by PCR targeting the 16S rRNA gene with prevalence of 40% [14]. The higher prevalence was attributed to their wild living conditions [14]. More recent studies in Europe have reported an upward trend of *Leptospira* infections attributed to climatic changes that results in wetter conditions that promote prolonged survival of the *Leptospira* bacteria in the environment and change in the herd management practices from indoor intensive to extensive or semi-intensive with outdoor access aimed at improved animal welfare [15–17]. In Africa several prevalence studies have been carried-out providing evidence of occurrence of leptospirosis in animals. Several studies in Tanzania have reported on pig leptospirosis; a serological survey tested 100 pigs using the MAT test showed high percentage of pigs positive to *L*. *kirschneri* serovar Sokoine (41%) and to *L. borgpetersenii* serovar Kenya (27%) [18]. Another cross-sectional survey tested pig sera using MAT in Morogoro municipality, reported an overall prevalence of 4.42%. Of the positive samples this study reported high proportions against *L. interrogans* serovar Ballum at 47%, *L. interrogans* serovar Icterohaemorrhagiae at 41% and *L. interrogans* serovar Pomona at 12% [19]. Porcine leptospirosis results in economic losses in pig farms due to fetal death, abortion, infertility and birth of weak piglets, subfertility as evidenced by reduced litter sizes has also been reported [20].

Globally, human leptospirosis cases have been estimated at about one million cases annually [21] resulting in the loss of 2.9 million Disability-Adjusted Life Years (DALYs) per annum [22]. The International Leptospirosis Society further estimates the incidence of severe human leptospirosis at 350,000–500,000 cases annually though this is maybe an underestimate due to lack of a notification system or since notification is not mandatory in most countries [5, 23, 24]. The burden of human leptospirosis is expected to rise with demographic shifts and climate change that result in heavy rainfall and flooding [25–27] resulting in loss of man hours and costs associated with medication in cases of chronic sequelae [28–31].

Leptospirosis is listed as one of the priority zoonotic diseases in Kenya based on a five point scoring criteria using the One Health Zoonotic Disease Prioritization (OHZDP) tool [32]. Earlier studies in Kenya in livestock focused on cattle, sheep and goats. A study by D’Souza [33] reported a 41% prevalence by MAT in a nation-wide serological survey of bovine leptospirosis [33]. Another nation-wide serological survey in goats reported a sero-prevalence of 16.2% by MAT [34]. A serological survey in Nyandarua and Turkana showed *Leptospira* antibodies by MAT were present in both regions in livestock and humans [35]. In Nyandarua, the study reported a prevalence of 49% in cattle and 55% in sheep and goats while in Turkana there was a prevalence of 44% in cattle and 24% in sheep and goats [35]. The higher prevalence in Nyandarua was attributed to the wetter climatic conditions compared to Turkana that may promote the survival of *Leptospira* bacteria in the environment [35]. A study among slaughterhouse workers in western Kenya using a commercial ELISA kit reported a seroprevalence of antibodies to *Leptospira* at 13.4% [36]. Though data on the incidence of leptospirosis is lacking, increasing reports of outbreaks in several parts of the world suggest that it’s re-emerging as an important public health problem [24, 37]. There are no studies on leptospirosis in pigs in Kenya thus the role of pigs in the transmission of leptospirosis is not known. This study aimed to estimate the sero-prevalence of *Leptospira* antibodies, identify some of the circulating serovars and identify associated factors among the slaughter pigs.

## Results

### Slaughter pig demographic characteristics

A total of 252 pigs were sampled from seven selected slaughterhouses. In total, 88.9% (244/252) of the pigs sampled were indigenous breeds. Female pigs accounted for 55.6% (140/252) of the pigs sampled and 88.7% (220/252) were sourced from farms practicing the extensive (tethering/free range) production system. The pigs were bought from small-scale farmers with 91.3% (230/252) of them owning less than 5 pigs. In 79.8% (197/247) of the farms, the pigs were reared with other types of livestock species (Table 1 and Additional file 1).

**Table 1:** Characteristics of pigs, proportion of MAT results, sero-prevalence and associated odds ratios by their demographic characteristics, (Antibody titre cut-off > 40), Busia County, Kenya 2018 (*n* = 252)

Variable	Variable categories	N (%)	MAT positive n (%)	MAT negative n (%)	Prevalence (95% CI)	Odds ratio (OR) 95% CI
Sex of pig	Female	140 (44.4)	49 (35.0)	91 (65.0)	35.0 (27.1–43.5)	1.2 (0.7–2.2)
	Male	112 (55.6)	34 (30.4)	78 (69.6)	30.3 (22.0–39.8)	Ref
Age category	Mature	110 (43.7)	45 (40.9)	65 (59.1)	40.9 (31.6–50.7)	1.9 (1.1–3.3)
	Young	142 (56.7)	38 (26.8)	104 (73.2)	26.8 (19.7–34.8)	Ref
Breed	Exotic/Crosses	28 (11.1)	10 (35.7)	18 (64.3)	35.7 (18.6–55.9)	1.2 (0.5–2.8)
	Indigenous	224 (88.9)	73 (32.6)	151 (67.4)	32.6 (26.6–39.2)	Ref
Body condition score	Poor/emaciated	45 (17.9)	20 (44.4)	25 (55.6)	44.4 (29.6–60.0)	1.7 (0.8–3.4)
	Good/very good	207 (8.2.5)	63 (30.4)	142 (69.6)	30.4 (24.2–37.2)	Ref
Herd size at the farm of origin	5+ pigs	22 (8.7)	11 (50.0)	11 (50.0)	50.0 (28.2–71.8)	2.2 (0.8–5.9)
	< 5 pigs	230 (91.3)	72 (31.3)	158 (68.7)	31.3 (25.4–37.7)	Ref
Production system	Extensive	220 (88.7)	71 (32.3)	149 (67.7)	32.3 (26.2–38.9)	0.8 (0.4–1.9)
	Intensive	28 (11.3)	12 (42.9)	16 (57.1)	42.9 (24.5–62.8)	Ref
Other types of livestock	Yes	197 (79.8)	72 (36.5)	125 (63.5)	36.5 (29.8–43.7)	2.3 (1.0–4.5)
	No	50 (20.2)	10 (20.0)	40 (80.0)	20 (10.0–33.7)	Ref
Presence of kidney white spots	Yes	21 (8.3)	7 (33.3)	14 (66.7)	33.3 (14.6–57.0)	1.0 (0.3–2.8)
	No	231 (91.7)	76 (32.9)	155 (67.1)	32.9 (26.9–39.4)	Ref
Sub county of origin	Butula	59 (23.4)	19 (32.2)	40 (67.8)	32.2 (20.6–45.6)	1.2 (0.3–5.9)
	Funyula	26 (10.3)	7 (26.9)	19 (73.1)	26.9 (11.6–47.8)	0.9 (0.2–5.3)
	Matayos	81 (32.1)	27 (33.3)	54 (66.7)	33.3 (23.2–44.7)	1.3 (0.3–6.0)
	Nambale	72 (28.6)	26 (36.1)	46 (63.9)	36.1 (25.1–48.3)	1.4 (0.4–6.8)
	(Butere/Matungu/Teso south)	14 (5.6)	4 (28.6)	10 (71.4)	28.6 (8.3–58.1)	Ref
Slaughterhouse						
Butula	Bumala	48 (19.1)	16 (33.3)	32 (66.7)	33.3 (20.4–48.4)	0.7 (0.4–1.6)
	Butula	18 (7.1)	3 (16.7)	15 (83.3)	16.7 (3.6–41.4)	
Funyula	Funyula	24 (9.5)	9 (37.5)	15 (62.5)	37.5 (18.8–59.4)	1.1 (0.4–3.1)
Matayos	Mundika	74 (29.4)	24 (32.4)	50 (67.6)	32.4 (22.0–44.3)	0.9 (0.4–1.8)
Nambale	Mungatsi	14 (5.6)	6 (42.9)	8 (57.1)	42.9 (17.7–71.1)	Ref
	Nambale	59 (23.4)	21 (35.6)	38 (64.4)	35.6 (23.6–49.1)	-
	Tanga-kona	15 (6.0)	4 (26.7)	11 (73.3)	26.7 (7.8–55.1)	-

Data are the number (%) pigs sampled, number (%) of leptospirosis positive pigs, number (%) of leptospirosis negative pigs, prevalence of leptospirosis seropositivity with their 95% confidence interval and odds ratios and their 95% confidence interval stratified by demographic characteristic and location. A pig serum sample was considered positive for leptospirosis when the endpoint titre was equal or more than 40 (MAT titre ≥1:40) against at least one serovar

*Ref* reference group

### Serological analysis

Of the 252 sera tested by MAT, 32.9% (95% CI 27.2–39.1%) tested positive for *Leptospira* antibodies to at least one serovar using a cut-off titre > 40. The apparent sero-prevalence for specific serovars were 21.4% (54/252) for *L. interrogans* serovar Lora; 5.2% (13/252) for *L. borgpetersenii* serovar Kenya; 3.6% (9/252) for *L. kirschneri* serovar Sokoine; and *L. interrogans* serovar Bataviae (Table 2). The apparent seroprevalence in each subcounty was 37.5% (95% CI 18.8–59.4%) in Funyula; 35.2% (95% CI 25.3–46.1%) in Nambale; 32.4% (95% CI 22.0–44.3%) in Matayos; and 28.8% (95% CI 18.3–41.4%) in Butula.

**Table 2:** Seroprevalence of *Leptospira* serovars and serogroups by microscopic agglutination test (titer > 1:40) among 83 positive samples in Busia County, Kenya

Genomospecies	Serogroup	Serovar	Positive (n)	Prevalence (%)	95% CI
L. santarosai	Hebdomadis	Hebdomadis	1	0.4	0.01–2.2
*L. interrogans*	Bataviae	Bataviae	9	4.8	1.7–6.7
	Pomona	Pomona	2	0.8	0.1–2.8
	Australis	Lora	54	21.4	16.5–27.0
L. kirschneri	Grippotyphosa	Grippotyphosa	8	3.2	1.4–6.2
	Icterohaemorrhagiae	Sokoine	9	3.6	1.7–6.7
*L. borgpetersenii*	Ballum	Kenya	13	5.2	2.8–8.7
	Sejroe	Sejroe	1	0.4	0.01–2.2

Data are number of samples *Leptospira* positive (cut-off titer > 1:40), the serovar specific prevalence and their 95% confidence interval. MAT end-point titre was defined as the highest dilution at which ≥50% of leptospires were still agglutinate

Out of the 252 pigs tested, the apparent seroprevalence among female pigs was 35% (95% CI 27.1–43.5%) compared to 30.4% (95% CI 22.0–39.8%) among male pigs. According to age category, the apparent seroprevalence among mature pigs was 40.9% (CI 31.6–50.7%) compared to 26.8% (CI 19.7–34.8%) in young pigs while in exotic/cross breeds the apparent seroprevalence was 35.7% (95% CI 18.6–55.9%) compared to 32.6% (CI 26.6–39.2%) in indigenous pigs (Table 1). The level of seropositivity by slaughterhouse showed varied from 42.9% (6/14) in Mungatsi to 16.7% (3/18) in Butula slaughterhouse (Table 1). Of the 252 sera tested, 6.8% (17/252) had relatively high MAT titers (Table 3).

**Table 3:** Frequency of MAT titer of pig sera by serovar in Busia County, Kenya (*n* = 109)

Serogroup	Serovar	1:20	1:40	1:80	> 1:160
Hebdomadis	Hebdomadis	0	0	0	1
Bataviae	Bataviae	18	5	2	2
Pomona	Pomona	0	1	1	0
Australis	Lora	4	25	18	11
Grippotyphosa	Grippotyphosa	2	5	2	1
Icterohaemorrhagiae	Sokoine	5	7	1	1
Ballum	Kenya	10	8	4	1
Sejroe	Sejroe	0	0	1	0

Data are the number of samples *Leptospira* reactive (cut-off titer > 1:20) by serovar

### Factors associated with *Leptospira* infection

There was no significant difference between seropositivity in female pigs compared to male pigs (OR 1.2, 95% CI 0.7–2.2) (Table 1). Mature pigs were significantly more likely to be seropositive compared to young pigs (OR 1.9, 95% CI 1.1–3.3). There was no association between breed and *Leptospira* seropositivity with exotic/cross breed pigs having OR 1.2 (95% CI 0.5–2.8) compared to indigenous breeds. The herd size at the farm of origin was not associated with *Leptospira* seropositivity with pigs being raised in a farm with more than 5 pigs having OR 2.2 (95% CI 0.8–5.9) compared to farms with 5 or less pigs. However, being raised with other livestock species was associated with *Leptospira* seropositivity (OR 2.3; 95% CI 1.0–4.5).

## Discussion

This study reports an apparent seroprevalence (32.9%) of leptospirosis in slaughter pigs in local slaughterhouses in a small holder livestock production system in the Lake Victoria basin, western Kenya. The study raises occupational health concerns that slaughterhouse workers are at risk of exposure to leptospires during their daily work routine. This is further compounded by the poor use of personal protective equipment among the slaughterhouse workers in the study area [38].

To the best of our knowledge, this is the first study demonstrating anti-*Leptospira* antibodies in pigs in Kenya. In the East Africa region a few studies have been conducted in Tanzania on porcine leptospirosis. A serological survey among a 100 pigs in Morogoro using the MAT test showed a high percentage of pigs positive for *L*. *kirschneri* serovar Sokoine (41%); *L. borgpetersenii* serovar Kenya (27%) and L. *kirschneri* serovar Grippotyphosa (22%) with MAT cut-off set at titres > 1:20 [18]. The lower cut-off may explain the higher estimate reported. The detection of antibodies against serovars Sokoine, Kenya and Grippotyphosa in the former study is similar to our findings suggesting that these serovars are a common cause of leptospirosis in pigs in East Africa. The study by Kessy et al. [19], reported a sero-prevalence of 4.42% in pigs in Morogoro, which was much lower than the prevalence in our study. A study in Morogoro municipality on pig production reported that over 94% of farmers confine their pigs [39]. The reasons for confinement were to avoid conflict with neighbors for religious reasons and local government regulations. We hypothesize that confinement inadvertently protected the pigs from infections and the external environment and this could explain the lower prevalence reported [39]. The study by Kessy et al. [19], also used a higher cut-off (> 1:160) compared to a cut-off (> 1:40) in our study. While the lower cut-off results in a higher prevalence, it ensured both recent and chronic infections normally characterized by low antibodies titres were reported as positive, an approach common in prevalence studies [33]. At a cut-off > 1:160, our study shows sero-prevalence of 6.8% (17/252) which is only slightly higher than the 4.42% reported by Kessy et al.*,* suggesting the infection rates in the two studies could be similar. Further, the high titers (> 1:160) could suggest some of the pigs sampled had active *Leptospira* infection.

Our study found the sero-prevalence among females (35%, 95% CI 27.1–43.5%) was slightly higher than among male pigs (30.3%, 95% CI 22.0–39.8%). This finding was similar to reports in fattening pigs in Vietnam, that showed the sero-prevalence among female pigs was higher compared to males [12]. Even though this association was not statistically significant, some studies have suggested venereal transmission occurs in leptospirosis and these could explain the higher prevalence in females [40]. Mature pigs (OR 1.9, 95% CI 1.1–3.9) compared to the young pigs were more likely to be seropositive, this finding compares with other studies that have suggested the difference to be attributed to increased exposure over time [12, 41].

Pigs being raised in a farm with other livestock species (OR 2.3; 95% CI 1.0–4.5) were more likely to be seropositive suggesting the possibility of transmission between multiple animal species [41]. A study on bovine leptospirosis in Brazil, reported co-grazing of cattle and other livestock species especially pigs was associated with bovine leptospirosis seropositivity [42]. Similar findings have been reported in three case reports of leptospirosis outbreaks in South Africa where infections occurred in mixed livestock species farming units [43]. In the first case report in Mpumalanga Province, 17% (9/52) of the pigs sampled in an intensively farmed 300-sow unit had positive titres by MAT. In the same farm, 52% (88/170) of the cattle and 1.3% (2/153) of the sheep tested positive. In the second case report in a 350-sow unit in the Bronkhorstspruit district, Gauteng Province reported 10.5% (4/38) of pigs sampled had high titres (median titre = 1/2560) to *L. interrogans* serovar Pomona. Cattle, sheep and dogs on the same farm were also infected with serovar Pomona. The third case report in Free State was an outbreak in a 250-cow Jersey herd with a 60-sow unit on the farm. At the herd level, 50% (104/204) cattle had MAT titres equal to and over 1:100 against serovars Icterohaemorrhagiae, Pomona and Bratislava. Pigs tested on the same farm showed positive titres in 45% (9/20) of the pigs against serovars Pomona and Bratislava [43]. A study in bovine leptospirosis in Tanga found that presence of sheep and goats on the farm were strongly associated with bovine seropositivity [44]. These reports support our findings that suggest an association between porcine seropositivity and the presence of other livestock species on the farm and this may be due to transmission between different livestock species.

Serological testing for leptospirosis using the gold standard MAT utilizes live cultures of *Leptospira* isolates of locally circulating serovars or serovars from reference laboratories [18]. As expected, our study demonstrated a higher prevalence against *Leptospira* serovars that have been previously isolated in the East African region. This is consistent with other research in the region [18]. The apparent prevalence of the locally isolated serovars were, Lora (21.4%), Kenya (5.2%), Sokoine (3.6%), and Grippotyphosa (3.2%) while apparent prevalence to the reference serovars were, Sejroe (0.4%), Pomona (0.8%), and Hebdomadis (0.4%). However, reference serovar Bataviae had a high apparent prevalence of 4.8%. Similarly the study by Kessy et al.*,* in Morogoro, Tanzania reported a low prevalence of serovar Pomona [19].

The serovar Lora used in the MAT panel was isolated from rodents in Tanzania [18]. This high prevalence of a rodent adapted serovar suggests transmission between the rodent maintenance host and pigs in the study area. A spatial ecology study in the Western Kenya region has reported pigs spend 47% of the time outside the owners farm and roam many kilometers scavenging for food [45]. This free-range system increases the risk of the pig acquiring zoonotic infections including leptospirosis due to extensive interaction with the environment and other maintenance animal hosts [45, 46]. A study in southern Sweden among outdoor reared pigs similarly reported the highest prevalence of antibodies to a local mouse strain (mouse 2A) [47]. The role of rodents as a source of infection of leptospirosis among domestic pigs has also been suggested by research in Vietnam [12, 41]. Previous research in Kenya has demonstrated the presence of pathogenic leptospires in rodents in both urban (18.3%) [48] and rural settings (41.8%) [49]. The role of rodents in the epidemiology of leptospirosis in Kenya requires further investigation.

There was limited information on the circulating serovars in the region, thus the serovars included in the test panel were selected based on studies conducted in the East African region [18]. It is likely that there are other serovars circulating in the area that were not included in the panel and could have led to an underestimation of the prevalence. Further studies to isolate *Leptospira* spp. circulating in Kenya and the East African region which can be stocked in the reference laboratories will go a long way in increasing the understanding of leptospirosis in the region. The MAT is relatively serovar-specific but cross reactions occur between related serovars particularly those in the same serogroup thus overestimate the prevalence. There is also evidence of seasonality between the wet and dry months of the year with a higher prevalence being reported during the wetter months [47]; our study was conducted during the dry months in the Lake Victoria basin region. These limitations are likely to have resulted in the under estimation of the sero-prevalence in this study. Slaughterhouse sampling strategy also meant that we lacked sufficient data on husbandry practices to allow for assessment of more risk factors that could increase or reduce exposure of pigs to leptospires. The study was unable to determine the impact of leptospirosis on the pig population in Busia County such as infertility and abortions but this needs to be established to understand the potential economic losses associated with this disease.

A previous study in the same area reported the seroprevalence among slaughterhouse workers at 13.4% demonstrating that leptospirosis is an important zoonotic disease in this population [36]. A similar study in the Tanga region in Tanzania among different occupational groups reported seroprevalence of 19.4, 18.1 and 17.1% in livestock farmers, veterinary/meat inspectors and abattoir workers respectively [50]. Several risk factors among the high risk groups have been identified including hygiene practices (hand washing during and after work, wounds/injuries, and eating and smoking during slaughter operations) that lead to exposure to leptospires [38, 51, 52]. These findings demonstrated that a significant proportion of people working closely with livestock are exposed to pathogenic *Leptospira*. The findings from these studies not only demonstrate the risk for slaughterhouse workers, but also people who handle pigs. Public-health interventions against leptospirosis should therefore target not only the high risk occupational groups but also the general population [50, 53].

## Conclusion

This study reports seroprevalence in slaughter pigs in slaughterhouses in western Kenya and highlights the potential occupational and public health risks. Prevention and control of the disease in pigs involves vaccination and a combination of effective strategies for farm biosecurity, good animal husbandry, and rodent control to prevent infection between the animal hosts and protect people [1, 38, 54]. Improvement in the husbandry practices (confinement and rodent control) and public health education among slaughterhouse workers and other high-risk groups on leptospirosis and the role of PPE use, personal hygiene might reduce the potential for transmission of leptospires.

## Methods

### Study area

**Fig. 1:**
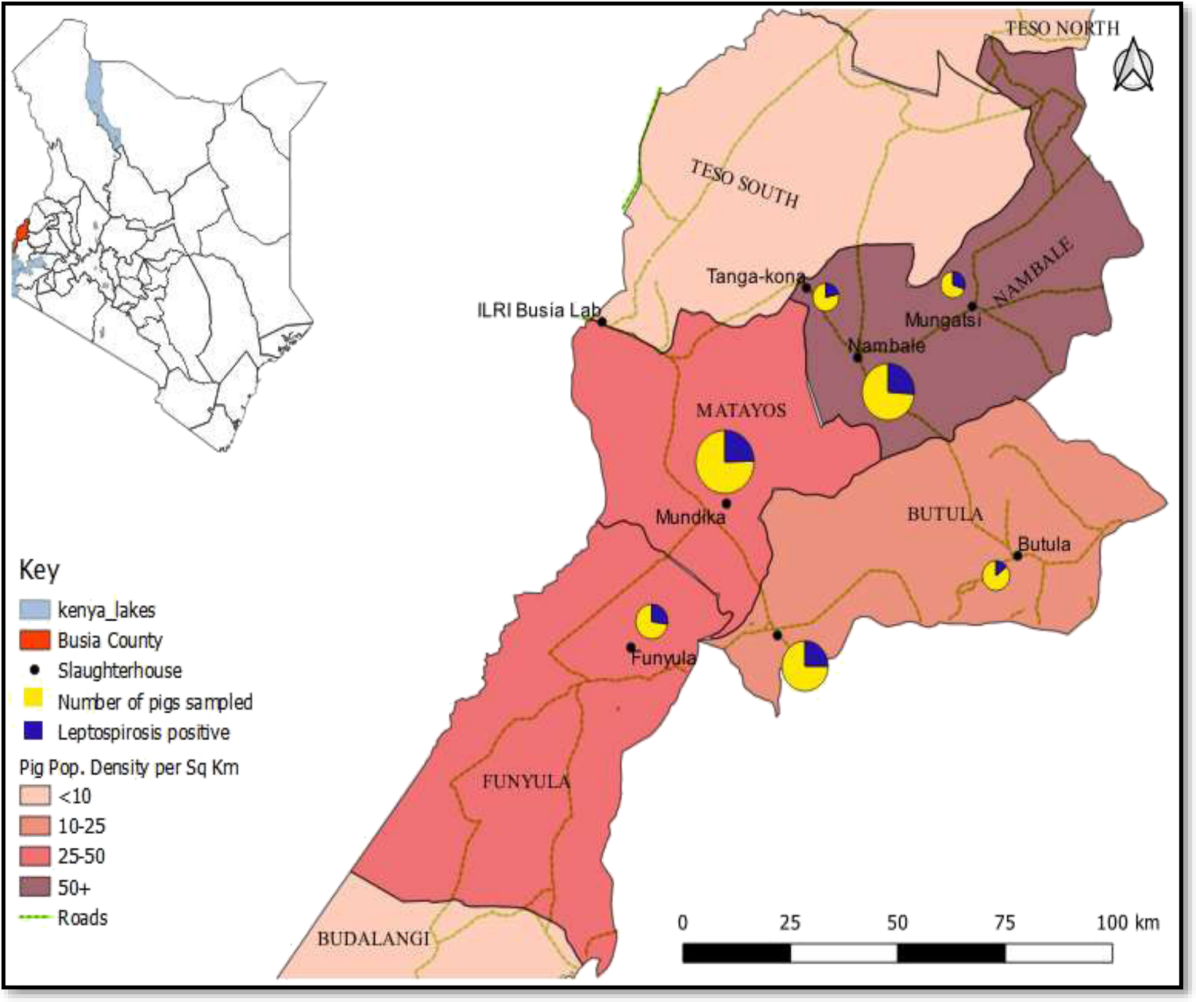
Map of the study area showing the locations of the slaughterhouses (pies), and the pig population densities (shading). The size of the pie indicates the number of pigs sampled with the dark-blue coloured wedge representing the number of leptospirosis positive pigs (Source: https://africaopendata.org/dataset/kenya-counties-shapefile 2019)

Busia County is located in the Lake Victoria Basin region on the border with Uganda and lies at latitude 0.4347°N and longitude 34.2422°E (Fig. 1). The county is predominantly rural with the main economic activity being crop and livestock subsistence agriculture. Pig production is an important economic activity in the study area with 26.2% of the national pig population found in western Kenya; of which 48,788 (55.5%) are in Busia County [55, 56]. Farmers in Busia County practice traditional free-ranging production systems where pigs are tethered or graze freely with only 4% of the pig rearing households providing improved housing [46]. The production system is also characterized by poor husbandry practices, biosecurity and disease control measures [45, 46].

### Study design

We conducted a cross sectional study involving pigs slaughtered at the licensed local slaughterhouses in urban centres within Busia County. The selected slaughterhouses were located in four subcounties (Nambale, Matayos, Butula, Funyula) where the pig population density was above 10 pigs per square kilometer [57].

### Study population

A list of all the slaughterhouses in the four sub counties was obtained from the County Director of Veterinary Services. Slaughterhouses that operated for at least 5 days in a week and slaughter at least five pigs daily were selected. The slaughterhouses were ranked according to the number of pigs slaughtered using the monthly average calculated from slaughter data reports from April to August of the last 2 years (2016/2017). Seven slaughterhouses that ranked highest based on the monthly average were selected. The geo-coordinates of each of the selected slaughterhouses were recorded using a Global Positioning System (GPS) unit and assigned a unique identification number.

The study population was slaughter pigs presented between May and July 2018 at the 7 selected category C slaughterhouses (facilities that slaughter pigs to be consumed in the immediate locality) [58] in Busia County.

All pigs presented at the selected slaughterhouses during the study period and whose owners consented were eligible to be part of the study.

### Sample size

A minimum sample size of 195 pigs was calculated using the Cochran formulae [59] with the following assumptions: seroprevalence of 4.42% [19], significance at *p* = 0.05, confidence level of 0.95 and design effect of 3.

### Sampling procedures

Systematic random sampling method was used. The minimum sample of 195 pigs was divided proportionate to size among the seven selected slaughterhouses based on the monthly average of pigs slaughtered calculated using data from the April to August period of 2016 and 2017. In each slaughterhouse, the monthly average of pigs slaughtered was divided by the allocated sample to get the sampling interval *(K*^*th*^*).* A random number (between 1 and *K*^*th*^*)* was selected as the starting point by randomly picking a hand-written piece of paper from a bucket and the sampling interval used thereafter to continue with the sampling until the required number of pigs was reached. To avoid calculating the sampling interval every time, pigs presented for slaughter were listed continuously through-out the study period in each slaughterhouse. After identifying the pig from which to collect biological samples, consent was sort from the slaughterhouse worker/trader. When the pig trader agreed to participate in the study, he/she signed a consent form. If the pig trader declined the next pig on the list was selected and consent sort. Animal information was collected using a questionnaire that was labelled using a unique serial number.

Variables collected included; the locality where the pig was purchased, breed, sex, age category, body condition score, presence of other livestock species at the farm of origin, number of pigs at the farm of origin, production system at the farm of origin and presence of kidney lesions at post mortem.

### Sample collection and processing

The identified pig was restrained using a snare, and 10 ml of whole blood was collected from the cranial vena cava using a 10 ml plain vacutainer tube and an 18-gauge needle (Becton, Dickinson and Company). The sample was labelled immediately with a unique number linking the sample to the questionnaire administered to the slaughterhouse worker/trader. All samples were allowed to clot at ambient temperature and transported in a cool box with ice packs to the ILRI Busia field laboratory. Serum was obtained from the clotted blood by centrifugation at 3000 RPM for 15 min and aliquoted into labelled cryogenic tubes and preserved at − 40 °C. The samples were couriered on dry ice to the Sokoine University of Agriculture, Pest Management Centre, Morogoro, Tanzania for serological analysis. The samples were maintained at − 20 °C during the period of serological analysis.

### MAT antigens selection and preparation

Live *Leptospira* antigens representing eight serogroups recommended for use in *Leptospira* testing in the East Africa region were used for screening the collected sera [18]. Four reference serogroups Hebdomadis (*Leptospira santarosai* serovar Hebdomadis), Bataviae (*L. interrogans* serovar Bataviae), Sejroe (*L. borgpetersenii* serovar Sejroe) and Pomona (*L. interrogans* serovar Pomona) which had been initially sourced from the WHO Reference Laboratory at the Royal Tropical Institute (KIT), Amsterdam, Netherlands. The other four were local isolates belonging to serogroups Grippotyphosa (*L. kirschneri* serovar Grippotyphosa), Icterohaemorrhagiae (*L. kirschneri* serovar Sokoine), Australis (*L. interrogans* serovar Lora) and Ballum (*L. borgpetersenii* serovar Kenya) maintained at the Pest Management Centre, Morogoro, Tanzania (Table 4). The live antigens were grown in fresh Ellinghausen and McCullough medium-modified by Johnson and Harris (EMJH) (Difco-USA) supplemented with *Leptospira* enrichment and 5-fluorouracil for 5 to 7 days until they reached a density of 3 × 10^8^ leptospires/ml based on the MacFarland scale, according to the guidelines of WHO/FAO/OIE Collaborating Centre for Reference and Research on Leptospirosis of the Royal Tropical Institute, Amsterdam, Netherlands. A loop-full of the stock culture was examined by dark field (DF) microscope to confirm the presence of viable leptospires and the absence of contamination.

**Table 4:** Serovars of *Leptospira spp*. used as live antigens in the MAT panel

Genomospecies	Serogroup	Serovar	Strain type
*L. santarosai*	Hebdomadis	Hebdomadis	Reference strain from KIT^a^
*L. interrogans*	Bataviae	Bataviae	Reference strain from KIT^a^
	Pomona	Pomona	Reference strain From KIT^a^
	Australis	Lora	Local isolate (Rodent) - Tanzania
*L. kirschneri*	Grippotyphosa	Grippotyphosa	Local isolate (Cattle) - Tanzania
	Icterohaemorrhagiae	Sokoine	Local isolate (Cattle) – Tanzania
*L. borgpetersenii*	Ballum	Kenya	Local isolate (Rodent) – Tanzania
	Sejroe	Sejroe	Reference strain from KIT^a^

^a^*Leptospira* strains from WHO/OIE Reference Laboratory - Royal Tropical Institute (KIT), Amsterdam, Netherlands, maintained at Sokoine University of Agriculture

### Antibody detection

The MAT test was used to demonstrate *Leptospira* antibodies in pig sera as described in the manual for International Course on Laboratory Methods for the Diagnosis of Leptospirosis [60]. Briefly, 10 μl of the sera were mixed with 90 μl phosphate buffered saline (PBS) in U-bottom microtiter plates to obtain 100 μl (1:10 dilutions). Serial serum dilutions were prepared in subsequent wells with 50 μl of PBS by pipetting 50 μl of the sera-PBS mixture into them. An aliquot of 50 μl of the fully-grown serovars in EMJH medium was added to the sera (final dilution 1:20) in the microtiter plate wells and mixed gently and then incubated at 30 °C for 2 h. The serum antigen mixture was then visualized by DF microscopy for the presence of agglutination and the titres recorded. MAT end-point titre was defined as the highest dilution at which ≥50% of leptospires were still agglutinated [61]. A serum sample was considered positive for seroreactivity when the endpoint titre was equal or more than 40 (MAT titre ≥1:40) against at least one serovar. Positive samples were further diluted to titres of 1:5120 to detect the end point titres. This was compared to the negative control containing 50 μl PBS mixed with an equal volume of the antigen in liquid EMJH (dilution 1:2). Serial dilutions of serovar-specific hyper-immune rabbit serum was tested with each serovar and the end-point titre established; this was used as a positive control.

### Statistical analysis

Data was stored using Microsoft Office Excel® 2013 (Microsoft Corp, Redmond, WA, USA) and analysed using Epi Info™7.1.4.0 (CDC, Atlanta, GA, USA) software. Proportions for categorical variables were calculated. The apparent prevalence estimates and their 95% CI were calculated using Epi Info7. Bivariate analysis to test for association between the independent and dependent variables was done, odds ratios (OR) and their 95% CI were reported. The independent variables included the breed, sex, age category, weight, body condition score, production system, other types of livestock reared at the farm of origin and presence of kidney white spots at postmortem and MAT test results as the outcome variable. Variables whose association with the outcome variable had a *P*-value of < 0.05 were considered to be statistically significant. The GPS information was used to map the location of the slaughterhouses using QGIS version 3.6.3 (ESRI, Redlands, California, USA) with base layers obtained from open data source (https://africaopendata.org/dataset/kenya-counties-shapefile). The number of sampled and positive cases by slaughterhouse were represented as pie charts on the map.

## Supplementary information

**Additional file 1.** Busia slaughter pigs leptospirosis data on pig demographic and husbandry practices characteristics and microscopic agglutination test results.

## Data Availability

All data generated or analysed during this study are included in this published article [and its supplementary information files].

## Abbreviations

°C: Degrees Celsius
CI: Confidence interval
DALY’s: Disability-Adjusted Life Years
DF: Dark field microscopy
ELISA: Enzyme-linked Immunosorbent Assay
EMJH: Ellinghausen, McCullough, Johnson and Harris
FAO: Food Agricultural Organization
GPS: Global Positioning system
ILRI: International Livestock Research Institute
KIT: Royal Tropical Institute
L: Leptospira
MAT: Microscopic Agglutination Test
OHZDP: One Health Zoonotic Disease Prioritization
OIE: World Organization for Animal Health
OR: Odds Ratio
PBS: Phosphate Buffered Saline
PCR: Polymerase Chain Reaction
RPM: Revolution per minute
rRNA: Ribosomal Ribonucleic Acid
SPMC: Sokoine Pest Management Centre
WHO: World Health Organization
